# Protocol of the Low Birth Weight South Asia Trial (LBWSAT), a cluster-randomised controlled trial testing impact on birth weight and infant nutrition of Participatory Learning and Action through women’s groups, with and without unconditional transfers of fortified food or cash during pregnancy in Nepal

**DOI:** 10.1186/s12884-016-1102-x

**Published:** 2016-10-21

**Authors:** Naomi M. Saville, Bhim P. Shrestha, Sarah Style, Helen Harris-Fry, B. James Beard, Aman Sengupta, Sonali Jha, Anjana Rai, Vikas Paudel, Anni-Maria Pulkki-Brannstrom, Andrew Copas, Jolene Skordis-Worrall, Bishnu Bhandari, Rishi Neupane, Joanna Morrison, Lu Gram, Raghbendra Sah, Machhindra Basnet, Jayne Harthan, Dharma S. Manandhar, David Osrin, Anthony Costello

**Affiliations:** 1 University College London, Institute for Global Health, London, UK; 2 Mother and Infant Research Activities (MIRA), PO Box 921, Thapathali, Kathmandu, Nepal

**Keywords:** Low birth weight, Pregnancy, Nutrition, Maternal health, Newborn, Cash transfer, Food supplement, Nepal

## Abstract

**Background:**

Low birth weight (LBW, < 2500 g) affects one third of newborn infants in rural south Asia and compromises child survival, infant growth, educational performance and economic prospects. We aimed to assess the impact on birth weight and weight-for-age Z-score in children aged 0–16 months of a nutrition Participatory Learning and Action behaviour change strategy (PLA) for pregnant women through women’s groups, with or without unconditional transfers of food or cash to pregnant women in two districts of southern Nepal.

**Methods:**

The study is a cluster randomised controlled trial (non-blinded). PLA comprises women’s groups that discuss, and form strategies about, nutrition in pregnancy, low birth weight and hygiene. Women receive up to 7 monthly transfers per pregnancy: cash is NPR 750 (~US$7) and food is 10 kg of fortified sweetened wheat-soya Super Cereal per month. The unit of randomisation is a rural village development committee (VDC) cluster (population 4000–9200, mean 6150) in southern Dhanusha or Mahottari districts. 80 VDCs are randomised to four arms using a participatory ‘tombola’ method. Twenty clusters each receive: PLA; PLA plus food; PLA plus cash; and standard care (control). Participants are (mostly Maithili-speaking) pregnant women identified from 8 weeks’ gestation onwards, and their infants (target sample size 8880 birth weights). After pregnancy verification, mothers may be followed up in early and late pregnancy, within 72 h, after 42 days and within 22 months of birth. Outcomes pertain to the individual level. Primary outcomes include birth weight within 72 h of birth and infant weight-for-age Z-score measured cross-sectionally on children born of the study. Secondary outcomes include prevalence of LBW, eating behaviour and weight during pregnancy, maternal and newborn illness, preterm delivery, miscarriage, stillbirth or neonatal mortality, infant Z-scores for length-for-age and weight-for-length, head circumference, and postnatal maternal BMI and mid-upper arm circumference. Exposure to women’s groups, food or cash transfers, home visits, and group interventions are measured.

**Discussion:**

Determining the relative importance to birth weight and early childhood nutrition of adding food or cash transfers to PLA women’s groups will inform design of nutrition interventions in pregnancy.

**Trial registration:**

ISRCTN75964374, 12 Jul 2013

**Electronic supplementary material:**

The online version of this article (doi:10.1186/s12884-016-1102-x) contains supplementary material, which is available to authorized users.

## Background

### Rationale

Our study assesses the relative impacts on birth weight and infant nutrition of an enhanced nutrition behaviour change strategy using Participatory Learning and Action (PLA), PLA plus an unconditional cash transfer, and PLA plus a food supplement, compared with standard antenatal care provided by current government programmes. The study will inform policymakers of whether each of the three interventions is more effective than current programme and what is the relative effectiveness of each intervention.

The main trial hypothesis is that an improved nutrition behaviour change strategy combined with a social transfer is an effective approach to improving birth weight and infant nutrition in rural south Asia. The justification for using community mobilisation through women’s groups as our behaviour change strategy is based on earlier research in rural areas of Nepal (Makwanpur district), Jharkhand and Orissa, India, Bangladesh and Malawi. These studies demonstrated the potential for participatory learning and action-based women’s groups to reduce neonatal mortality [1–4], and also maternal mortality in meta-analysis [5]. The intervention appeared equitable [6] and an economic evaluation found it to be cost effective [7]. Nonetheless, the trials assessed the impact of participatory women’s groups on mortality rather than nutritional status of pregnant women or low birth weight.

Low birth weight (LBW) is common in low-income countries such as Nepal, mainly representing intrauterine growth retardation and preterm birth, and is associated with outcomes including increased neonatal mortality and childhood stunting, adult chronic disease, and impaired cognitive function [8–12]. 60 % of neonatal deaths are in LBW infants [13].

We know of no trial that has assessed the impact of this approach on these outcomes, although others have used various approaches to providing dietary advice in pregnancy, with limited success [14].

In Dhanusha district, we previously helped local Female Community Health Volunteers to facilitate women’s groups which engaged with communities around issues of maternal and young child nutrition and postpartum care behaviours, as well as maternal and newborn health issues [15]. Our understanding of socio-cultural issues affecting good nutritional behaviours was developed through formative research, exploring preferences, taboos and beliefs about what is good for mothers [16].

The justification for assessing the impact of unconditional food supplements and cash transfers rests on the relative paucity of evidence for their impact in South Asia. Evidence that food supplementation can increase birth weight exists [17–21] and some limited evidence for the impact of cash transfers on birth weight has been found from large-scale cash transfer programmes in South America [21–24]. No study has compared the effectiveness of food supplementation versus cash or versus behaviour change alone.

## Methods

### Trial design

The study is a cluster randomised controlled trial in which 80 clusters are allocated to four arms, each of 20 clusters: (i) control receiving current Government of Nepal health services, (ii) enhanced Participatory Learning and Action nutrition behaviour change strategy for women in pregnancy (PLA), (iii) PLA plus a food supplement, and (iv) PLA plus an unconditional cash transfer. The cluster unit of randomisation is the Village Development Committee (VDC), with population size between 4000 and 9200 (mean 6150), drawn from Dhanusha and Mahottari districts in the plains of Nepal, bordering Bihar state, India. In order to reduce heterogeneity between clusters, we excluded VDCs that: were previous women’s group intervention areas; had large towns and municipalities; had hilly, forested or non-Maithili-speaking areas; along the East–west highway; and with population <4000 or >9200.

**Fig. 1:**
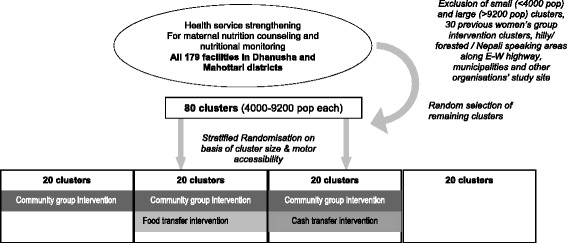
Trial design

The design is outlined in Fig. 1, together with the randomisation process.

### Setting

**Fig. 2:**
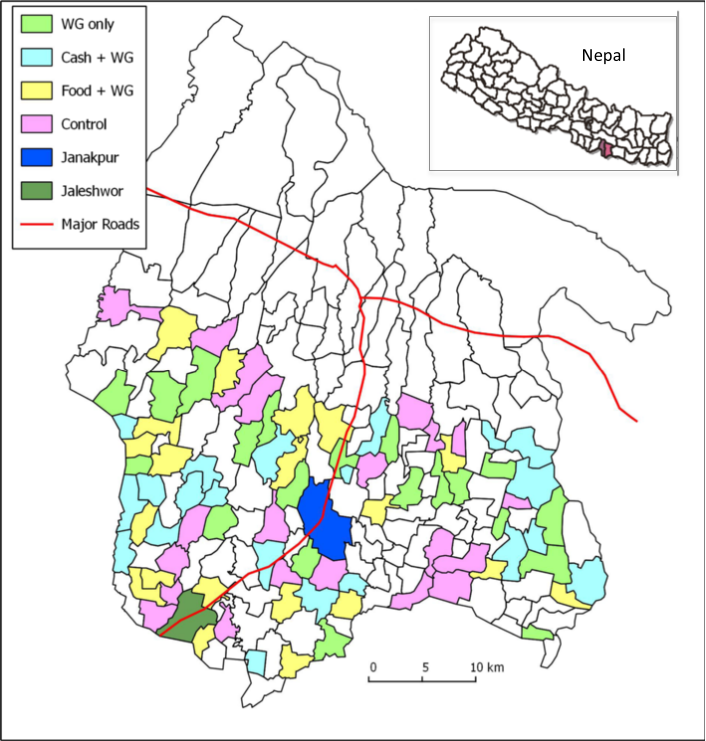
Map of Mahottari and Dhanusha districts showing study clusters and allocation

The study involves two contiguous districts, Dhanusha and Mahottari, in the central *Terai* (map in Fig. 2), with a combined population of 1.4 million [25] (>80 % in rural areas), and female literacy rates of 44 and 39 %, respectively [26]. This Maithili-speaking area of the central *Terai* is culturally and environmentally similar to large areas of Bihar, Uttar Pradesh, and India’s other northern plains areas, as well as areas of Bangladesh and Pakistan. Maithili women, especially young wives, are often required to remain in their homes out of sight of non-family members. Intra-household food allocation is particularly inequitable [27, 28] and levels of undernutrition in women and girls are high. Central *Terai* data from the Nepal DHS survey 2011 indicate that 26 % of women have body mass index (BMI) <18.5 kg/m^2^ and 14 % are below the 145 cm cut-off for short stature [29]. Data from the our previous trial population in Dhanusha in 2011 [15] indicate that 40 % have BMI <18.5 kg/m^2^ and 17 % have short stature. Child undernutrition is also high, with stunting at 41 %, underweight at 32 %, and wasting at 10 % [29].

Moderate-to-severe food insecurity in the Central *Terai* is about 20 % less common than in the more remote high-altitude areas of Western Nepal [29]. There may be more scope for cash transfers to contribute to micronutrient-rich diets in less poor socioeconomic groups, and more scope for a food supplement to be given to the pregnant woman in a household if other family members are less in need, providing a fair test of the transfers. In the poorest quintile, however, moderate to severe food insecurity is more prevalent. Studying the use of food and cash in these households will enable us to assess the potential impact of the transfers on infant nutrition amongst food insecure households.

The trial partners include University College London, Mother and Infant Research Activities (MIRA), Save the Children, World Food Programme and the Institute of Fiscal Studies, UK.

### Interventions

The study is designed with independent interventions and surveillance systems to as great an extent as possible. However, interventions depend on the surveillance system to generate pregnancy ID cards, which enable women to access transfers. In transfer clusters the women’s group intervention is integrated with the delivery of cash and food transfers, but otherwise interventions are managed independently of one another.

### Participatory Learning and Action women’s group enhanced nutrition behaviour change strategy intervention (PLA)

Every ward division of each VDC in Nepal has a Female Community Health Volunteer (FCHV), a frontline health volunteer tasked within the Government of Nepal (GoN) health system to conduct a monthly women’s group meeting, amongst other activities. In the past most of the groups met irregularly and usually without any specific agenda or activity, unless coupled with immunisation or vitamin-A supplementation programmes. Recently, however, the GoN community-based neonatal care package (CBNCP) is strengthening women’s groups in some districts. Based on previous success in revitalising these groups using Participatory Learning and Action (PLA) with interactive agendas for discussion [5, 30], we hypothesised that the groups would be an appropriate platform for a nutrition behaviour change strategy and for distribution of social transfers.

The 60 clusters in which the PLA is being implemented have 540 FCHVs and 539 women’s groups (WGs). One sparsely populated ward was merged with its neighbour. Formative studies of Female Community Health Volunteers (FCHVs) in Dhanusha and Mahottari showed that 60 % were illiterate and 67 % over 40 years of age, so we decided that a literate assistant would be required for each group. We recruited 539 literate incentivised volunteers called nutrition mobilisers (NMs), with a minimum education to 5th grade, to take responsibility for following the women’s group manual and keeping records of transfers and group activities. Both FCHVs and NMs receive small cash incentives of NPR400 ($4) per month for facilitating women’s groups and NPR 400 per month for attending orientation meetings, plus NPR 20 per sack of food and NPR 10 per envelope of cash delivered via home visits.

**Fig. 3:**
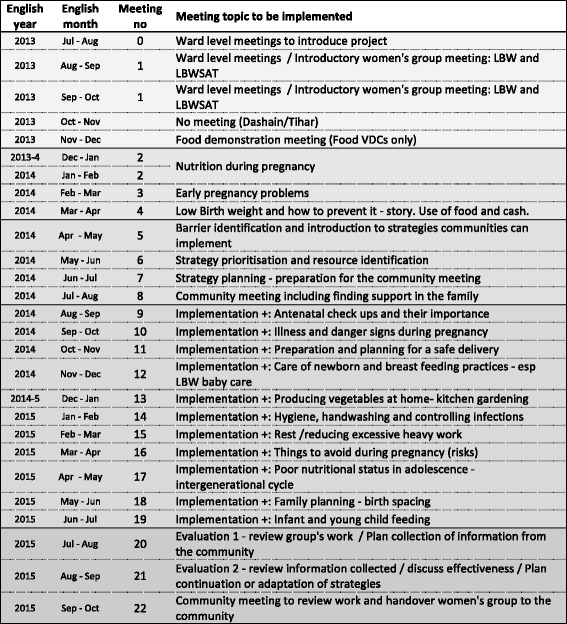
Schedule of women’s group meetings

Each women’s group follows a PLA cycle to mobilise the community for behaviour change. A pictorial meeting manual, with accompanying picture cards for each meeting, provides a monthly agenda. Formative research into local perceptions about food in pregnancy and baby size, food taboos, empowerment, experience from a previous cRCT about maternal and infant nutrition in Dhanusha district, and literature on risk factors for low birth weight informed the meeting content. Pre-existing tools from the GoN and other organisations working on nutritional interventions in Nepal (such as *Suahaara* and World Vision) were used or adapted where appropriate. A series of 22 meetings covers topics associated with maternal nutrition and the intergenerational cycle of under-nutrition, including foods needed for good health and exposures to avoid in pregnancy; pregnancy, delivery and postpartum danger signs and care-seeking; infection control and hygiene; importance of rest and family support; care of low weight infants; breastfeeding and infant feeding; disadvantages of adolescent pregnancies; birth spacing and family planning; and kitchen gardening. A recurrent theme on the importance of handwashing and hygiene runs throughout the meeting cycle. A full schedule of 22 meetings is provided in Fig. 3.

Prior to the intervention, which began in September 2013, 540 FCHVs and 539 NMs were trained by 30 Facilitation Field Coordinators (FFCs) in facilitation skills and potential barriers to good maternal nutrition, and the importance of rest and infection control in pregnancy. At each subsequent monthly orientation meeting, FFCs train the FCHVs and NMs on the women’s group manual agenda for the coming month using role-play, games and picture cards. FFCs visit the FCHV-NM pairs in their intervention wards on rotation to support them in facilitating group discussions, problem solving and providing ongoing training. FFCs aim to observe two-thirds of meetings and provide feedback on the facilitation and transfer distribution processes.

The FCHV/NM facilitator is encouraged to pose questions, enabling members to use existing knowledge and resources in answering them, debate current practices and the potential for change, and identify their own gaps in knowledge. Groups are encouraged to think about the key stakeholders in the intervention (for example, village leaders, politicians, mothers-in-law, health workers, husbands), and how to involve them in addressing barriers and increasing the proper use of the food and cash transfers. A similar intervention in Malawi found it beneficial to inform men of group activities relatively early in the meeting cycle [31].

Maternal nutritional problems are identified by discussion, games and stories over a series of meetings using pictorial aides, potential strategies are discussed, prioritised and then presented to the community at a ward-level meeting. Once community support has been enlisted, groups implement their strategies for a number of months. Strategies mainly involve raising awareness in the wider community through home visits to pregnant women who do not attend groups, playing a picture card game in the community, separate meetings to engage with mothers-in-law, male family members or adolescent girls, nutrition rallies on the importance of maternal nutrition, and showing pregnancy-related videos. Communities finally evaluate their strategies over 2 meetings and the groups are handed over to the community.

#### Design of the unconditional transfer interventions

**Fig. 4:**
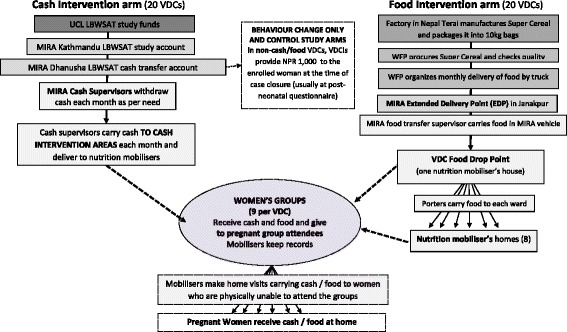
Food and cash transfer delivery system

Nutrition Mobilisers (NMs) distribute the food, supported by FCHVs and women’s group members. Figure 4 shows the transfer delivery system, which is similar in both food and cash transfer arms. Women’s group members assist the nutrition mobiliser in identifying beneficiaries early in their pregnancies and encourage them to come to the group to receive a monthly food transfer. If a woman is physically unable to come to the group in person (including if she is not allowed to attend by her family), the nutrition mobiliser takes the transfer to the pregnant woman’s home and explains the importance of eating well in pregnancy (using picture cards). In food transfer arms the NM also explains how to prepare the food and how much should be eaten per day. In the cash transfer arm the NM recommends food purchases and frequency over the month.

In order to qualify for a transfer a woman must have a valid pregnancy ID card and be pregnant. Each woman can receive a maximum of 7 monthly transfers. Recording of transfers at the time of delivery is done by NMs by collecting signatures or thumbprints and scanning QR codes of recipients (from their ID cards) using Android smartphones.

Group members and nutrition mobilisers check that all pregnant women in their ward, including women from the poorest and most marginalised households, receive an ID card and transfers. The nutrition mobilisers (and FCHVs if they able to help) are paid a monthly incentive for transfers and home visits conducted.

#### Food transfers

The food transfer comprises a 10 kg sack of Super Cereal provided monthly from 8 weeks gestation until birth. Super Cereal has been developed and tested by WFP and is being distributed elsewhere in Nepal to pregnant and lactating women and their young children. We conducted a formative study from September 2012 to January 2013 and found the cereal to be acceptable to pregnant women, particularly when consumed in the form of *haluwa* (a sweet dish prepared by roasting the flour in oil or ghee and adding water and sugar to make a thick paste) or as *roti* (unleavened flatbread). The nutrient content of Super Cereal is provided in Additional file 1. We recommend that women consume a Super Cereal ration equivalent to 150 g of dry flour per day so that, if needed, most of the micronutrient and additional energy requirements of pregnancy can be met from consuming only this food plus iron-folate supplements.

A 150 g intake of the supplement per day meets the total requirements for all nutrients except iron, folic acid (both of which we would expect to come from iron-folate supplements provided by the GoN via FCHVs), iodine (which should come from iodised salt consumption), zinc, copper and selenium. Slightly lower than recommended values are also found for retinol, thiamine, niacin, vitamins B2 and B6, and zinc in a 150 g serving of Super Cereal, but the shortfalls are small and may be less important than the bigger challenge of ensuring that each woman consumes her daily recommended amount. Women eat normal family foods in addition to the food supplement, which may include nutrient-rich green leafy vegetables, Vitamin A-rich fruits and vegetables, pulses, dairy products and sometimes meat and fish.

The Super Cereal is manufactured by a WFP-registered factory in Sunsari district (~100 km from the MIRA field site office in Janakpur), tested for quality by WFP, and delivered monthly to an “Extended Delivery Point” (EDP) food store, which meets stringent storage regulations provided by WFP for ventilation, vermin control and maximum stack height. Sacks of Super Cereal are delivered from the EDP to one Food Drop Point (FDP) per food cluster monthly or, when weather conditions render road access impossible, in bulk to cover the monsoon months. FDPs are also required to meet WFP storage standards and are inspected regularly. Any sacks of food suffering damage are destroyed. From the FDP, the food is carried by incentivised porters to nutrition mobilisers’ homes at around the time of the women’s group meeting. Supplies are usually stored in the home of the nutrition mobiliser or FCHV until the monthly group meeting when ID card-holders receive their food transfers and non-attendees get a home visit.

#### Unconditional cash transfer

The cash transfer is NPR 750 ($7) per month. Cash is collected from district headquarter banks by MIRA staff, and delivered to nutrition mobilisers in cash arm communities. A stringent security protocol is followed.

#### Purchasing power of the cash transfer

Formative data suggested that a transfer of cash could be very important to the poorest families in increasing access to nutrient-dense foods for pregnant women, but might be needed to feed the whole family, not just the pregnant woman [15].

In 2012, we used Save the Children Cost of Diet linear programming tool [32, 33] to estimate the minimum cost of a nutritionally adequate food basket using price data from local markets. The Nepal Rastra Bank [34] estimated that food price inflation varied from 10 % in 2007–8 to 16 % in 2008–9. Our own food price inflation estimates based on 66 food prices in Dhanusha district sampled in 2006, 2008 and 2009 indicate that average food prices in Dhanusha increased by 33 % between 2005 and 2008 and by 25 % between 2008 and 2009. Based on a 10 % per annum inflation rate (Nepal Rastra Bank consumer price index estimates [34]), the estimated cost of a nutritionally adequate diet for 2012 was NPR 57 a day or NPR 1767 per month.

The cash transfer should therefore cover a significant proportion of the necessary expenditure on micronutrient-rich items such as dairy products, meat, fish and eggs and fruits which the pregnant woman needs to eat to meet her minimum nutrient requirements.

We estimate that the food production costs NPR 70 per Kg, plus NPR 6 per Kg for transport making a total of NPR 760 per 10 Kg bag per month. We decided that the cash transfer should roughly match the value of the food transfer and set the cash transfer at NPR 750 per month, which is sufficient to buy 43 % of the cereal’s micronutrient value.

#### Home visiting

NMs provide home visits to women who are unable to attend groups. The NMs deliver food or cash and provide counselling on nutrition. NMs also visit women at home to encourage them to attend groups and FCHVs visit pregnant and postpartum women as part of their routine duties within the GoN health service. Home visiting by NMs, FCHVs and women’s group members to provide counselling and show picture cards to pregnant women was widely adopted as a group strategy. Structured home visiting by nutrition mobilisers through use of a nutrition counselling manual was originally planned, but was not feasible due to overburdening of the volunteer nutrition mobilisers and FCHVs.

#### Current government programmes available throughout the study districts

Women enrolled in all arms of the trial are able to access current government health service programmes which include antenatal care at local health facilities (a woman who makes 4 antenatal visits receives an incentive of NPR 400 at the time of her final visit), and visits from FCHVs in which they provide free iron-folate supplements from the fourth month of pregnancy onwards, and a single dose of anthelmintic. For perinatal care, services include a maternity incentive of NPR 500 for women who go to government (and certain participating private) health facilities for institutional delivery, and postnatal visits by FCHVs to distribute vitamin A and iron-folate. The Community-based Newborn Care Package (CBNCP) operates in Mahottari district only over the study period. This involves FCHVs making postnatal visits to check on neonates and provision of agendas for FCHVs to facilitate women’s group meetings.

#### Benefits to control clusters

In order to ensure that control clusters do receive some benefit from the project we strengthen government services in the two study districts (regardless of trial arm allocation). Using the Nepal National Maternal Newborn and Child Health (MNCH) communication strategy, we train health workers on tackling the inter-generational effects of undernutrition, diet in pregnancy and during lactation, rest during pregnancy, intestinal parasites and infections in pregnancy, maternal and newborn anthropometry, management of LBW infants through Kangaroo Mother Care, estimating BMI, BMI cut-offs for under- and over-nutrition and the risks of obesity.

#### Benefits to non-transfer women’s group and control arm participants

Because provision of social transfers could generate conflict and a lack of community cooperation in non-transfer areas and because participants give up their time for data collection, we compensate women for participating in the control and women’s group only arms of the trial with a cash transfer of NPR 1000. This is paid at the time of the 6–8 week postpartum interview or at the endpoint nutrition follow-up clinics.

#### Criteria for modifying allocated interventions

We aim not to modify the design of any interventions significantly over the duration of the trial unless realities on the ground and the preferences of the participating communities change significantly.

#### Process evaluation

**Fig. 5:**
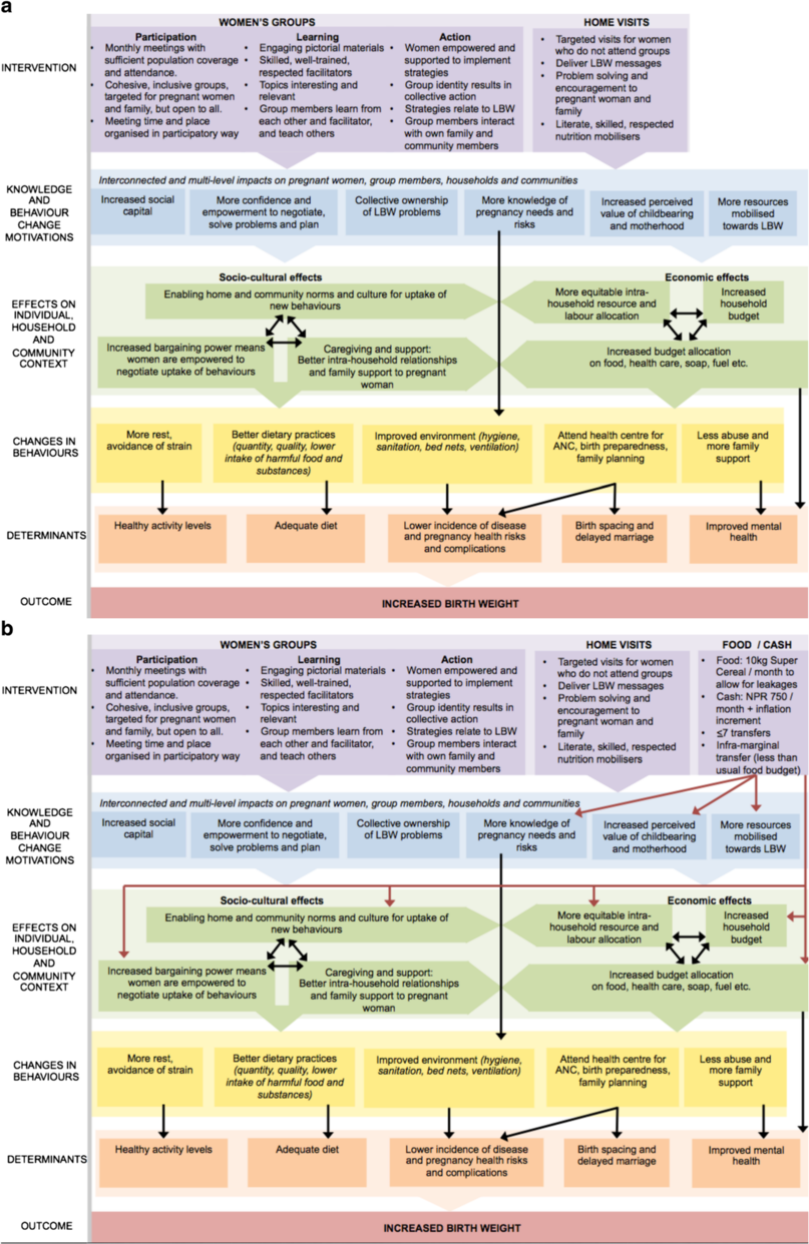
**a** Theory of Change for Participatory Learning and Action only. **b** Theory of Change for Participatory Learning and Action plus food or cash transfers

A detailed process evaluation of the three interventions using a realist approach [35] will describe the baseline and changing context in intervention and control clusters, implementation of the interventions, and explore mechanisms through which the interventions work or do not work. Our theory of change is shown in Fig. 5a and b, which consider risk factors for low birth weight and contextual factors that may impact upon intermediary outcomes and birth weight.

To understand context, we monitor women’s group status before interventions begin and in control areas, FCHV characteristics, and activities of community-based organisations and government programmes such as CBNCP and the “1000 Golden Days” nutrition programme. We track the progress of interventions through observation of monthly intervention team meetings and structured observation of one group per study arm. Using qualitative methods, we conduct focus group discussions with trial intervention field workers, including those distributing cash and food, and explore factors affecting how food and cash are being utilised in households and women’s access to and participation in the PLA intervention.

#### Strategies to improve adherence to intervention protocols, and procedures for monitoring adherence

Adherence to intervention protocols is improved by detailed process monitoring: observation of women’s groups by facilitation field coordinators (FFCs), including filling of an observation checklist on a smartphone for each meeting observed; monitoring of nutrition mobilisers to record details of delivery of transfers, women’s group attendance, home visits including topics covered and returned food transfers (due to food spoilage). Post-distribution monitoring is conducted by Save the Children fieldworkers who visit a random sample of women who receive transfers each month and fill in a questionnaire on a mobile phone.

### Participants

#### Eligibility to participate in interventions and surveillance

All married women between 10 and 49 years who have not had operative family planning, and whose husbands have not had vasectomy, are eligible for menstrual monitoring. In an August - November 2013 household census, 64 000 eligible women consented to this. Names and contact details are entered into pre-printed registers with a unique ID number and quick response (QR) code for each participant. These IDs are scanned with Android smartphones when women enter the trial and remain with them throughout. A woman may have more than one pregnancy in the study and each is identified with a pregnancy number suffixed to the woman ID. New women are added to the registers prospectively as they move into the clusters.

Any woman whom a ward enumerator identifies as having missed two menses in a row is eligible for a pregnancy test to enrol into trial interventions. In-migrating pregnant women who were not enrolled in menstrual monitoring at the time of the census are also eligible to enrol for interventions so long as they plan to be resident for at least 3 months.

In order to enrol and be followed up, a woman must have a positive pregnancy test or (in the case of a negative test in later pregnancy) look obviously pregnant. No limit has been set on gestational age at enrolment in order to avoid women providing inaccurate dates of Last Menstrual Period (LMP) in order to access transfers. We define permanent migration as a pregnant woman or mother moving out of the study area without any apparent plan to return within the entire follow-up period. A mother-child dyad is also considered lost to follow-up if information on a woman or her baby, or a relative representing them, cannot be found at the time of infant nutritional follow-up clinics.

#### Participant eligibility for inclusion in analyses

Participant eligibility for trial analyses depends on whether she has in-migrated as a (usually newlywed) permanent resident living with her husband’s family, or whether she is migrating temporarily to her parental or other relative’s home in order to access transfers. Women who enrolled in the census or are newlywed in-migrators living with their husband’s families (*sasural*) are “full trial participants”. Women who were not enrolled during the census and who have in-migrated to their parental (*maiti*) or ‘other’ home are “intervention only participants”.

Full trial participants are eligible for inclusion in the primary intention-to-treat analysis if they had the opportunity to receive >16 weeks exposure to an intervention during their pregnancy. In practice, this means inclusion of all full trial participants who enrolled during the intervention run-in period (13 Feb – 4 Jun 2014) or in the full trial surveillance period (5 Jun 2014 – 28 Feb 2015), who delivered after 5 Jun 2014, irrespective of their level of engagement with the intervention or their gestational age at enrolment. Multiple births, infants whose mothers have died, and infants with congenital abnormalities will be excluded from analyses.

Infants born to women who delivered before the start of the first endpoint nutrition follow-up clinics (20 June 2015) will be eligible for inclusion in endpoint weight-for-age Z-score (WAZ) analyses. Infants born after 20 Jun 2015 and infants born before interventions began (between 29 Dec 2013 and 12 Feb 2014) may be included in sub-analyses, but are excluded from primary analyses.

Our definition of loss to follow-up includes: a) women enrolled as pregnant who subsequently miscarry, b) stillborn infants, c) early neonatal deaths before birth weight can be taken, d) women who, once enrolled, move out of the project area and cannot be found.

#### Outcomes

The original primary outcome was birth weight, measured within 72 h after birth using infant weighing scales accurate to 10 g. As birth weights in the community are difficult to capture within 72 h (as found in a recent trial in Mumbai [36]) a second primary outcome of endpoint weight-for-age Z-score in infancy is collected through cluster nutrition clinics during June to October 2015.

**Fig. 6:**
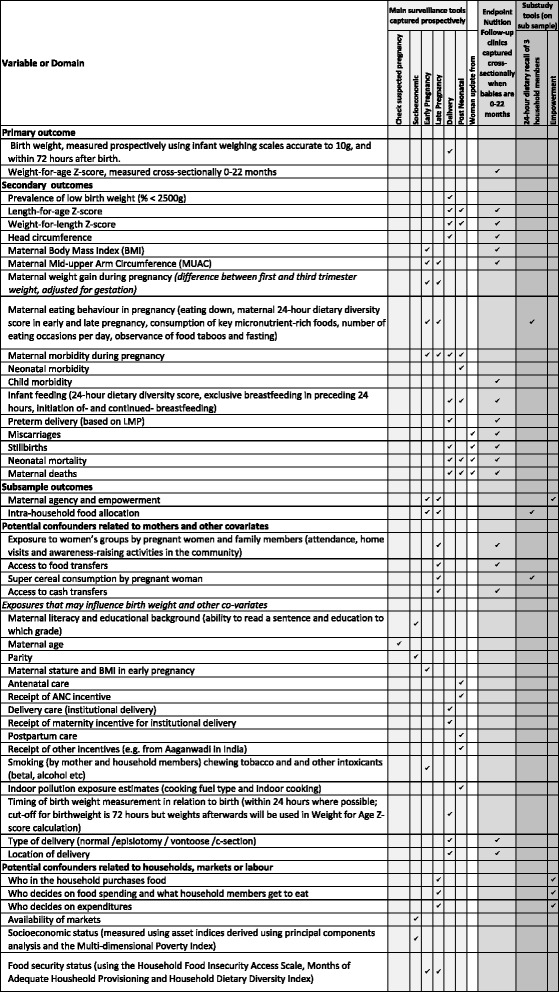
Trial outcomes, confounders and exposures to interventions

Primary and secondary outcomes, proposed confounder covariates and measures of exposure to interventions are summarised in Fig. 6. Secondary outcomes include maternal eating behaviour and weight gain in pregnancy; pregnancy, postpartum and neonatal illness; preterm delivery, miscarriage, stillbirth, and neonatal mortality. Secondary outcomes arising from endpoint nutritional follow-up, which may occur 0–22 months after delivery, include infant Z-scores of length-for-age, weight-for-length, infant head circumference, maternal BMI and mid-upper arm circumference (MUAC). Coverage of interventions by exposure to women’s groups, food or cash transfers, home visits, and group interventions in the community, adherence to recommended intakes of Super Cereal, and factors such as smoking and indoor pollution, micronutrient supplement intake, age and parity are measured.

#### Participant timeline

**Fig. 7:**
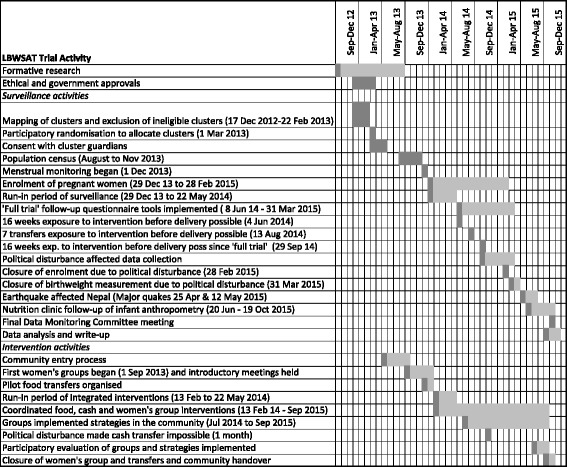
Trial timeline

Figure 7 shows the trial timeline. Formative research to inform the design of the study and development of electronic data collection systems began in September 2012. A population census ran from August to November 2013. Menstrual surveillance began 1 Dec 2013 and enrolment and follow-up of pregnant women on 29 Dec 2013. Piloting of food transfer delivery took place in Dec 2013 and Jan 2014. Integrated delivery of food and cash through women’s groups began on 13 Feb 2014. The ‘run-in’ of interventions ran from 13 Feb to 4 Jun 2014. Enrolled women delivering over this period had less than 16 weeks potential exposure to interventions. The full trial phase began on 5 Jun 2014 after 16 weeks of potential intervention exposure became possible. The full 7 ‘doses’ of transfers could potentially have been received amongst women delivering after 13 August 2014 (counting those enrolled in run-in). Sixteen weeks of exposure for women enrolled in the full trial became possible for those delivering after 29 Sept 2014.

Political disturbance severely disrupted trial surveillance activities (but affected interventions only slightly) from late September 2014 to January 2015 and this led to premature closure of surveillance due to lack of funds. Enrolment of pregnant women closed on 28 Feb 2015 and follow-up using routine trial surveillance systems, including capture of birth weight, closed on 31 March 2015. Interventions continue to run for women due to deliver between April and October 2015.

Two devastating earthquakes hit Nepal on 25 April and 12 May 2015. Although the study population was not severely affected, some disruption was experienced, especially to researchers based in Kathmandu.

In order to capture anthropometric outcomes for as many woman-infant pairs as possible after closure of surveillance, nutritional follow-up clinics are implemented to enable a one-off cross-sectional sample per cluster from 20 June to 19 October 2015.

#### Sample size

For 80 % power to demonstrate a difference between two study arms, where the underlying difference between arms is 50 g in mean birth weight (the primary outcome), we require at least 17 clusters per arm to contribute data and at least 163 births per cluster on average. We therefore randomised twenty clusters to each arm to allow up to 3 clusters to drop out from each arm. The calculations are based on comparisons between two arms, and apply to any pair of study arms. Table 1 summarises the number of clusters per arm to provide 80 % power to detect a significant difference between two study arms, assuming a standard deviation (SD) of birth weights within each study arm of 410 g, an intra-cluster correlation coefficient (ICC) of 0.01 and taking the standard two-tailed 5 % significance level. The design effect is 2.62 for clusters of 163 births. Due to difficulties obtaining the required number per cluster, but seeing little chance of cluster drop-out from the study, calculations were repeated during the trial to investigate the number required per cluster with no loss of clusters. With all 20 clusters per arm contributing outcome data, 111 birth weights per cluster (design effect 2.10) provide the same effective sample size as shown in the Table 1 and hence the same power as originally proposed. A target sample of 111 birth weights per cluster, 2220 per arm and 8880 in total, was adopted.

**Table 1:** Sample size for birth weight, at 80 % power, 5 % significance level, and intracluster correlation coefficient 0.01

Difference between arms, g	Effective sample size per arm	Number of VDCs per arm, 163 births per cluster	Number of VDCs per arm, 111 births per cluster
75	470	8	9
50	1056	17	20
25	4223	68	80

Power to rank arms for effect on birth weight (i.e. to see higher sample mean birth weight in the more effective arm) is good (>80 %) provided that the difference between arms is 15 g or more. A 25 g difference provides 92 % power, and 20 g provides 87 % power.

For the proportion of LBW infants, the sample size provides 82 % power to detect a reduction from the anticipated level of 27.5 % under current programmes to 22 % under one of the interventions, again at a two-tailed 5 % significance level. The ICC of 0.01, SD of 410 g and a LBW level of 27 % is based on 2011 MIRA Dhanusha data [37]. Our antenatal micronutrient supplementation trial in the same population showed a difference in birth weight of 77 g [38]. Pooled analyses suggest a combined effect of 22 g from multiple micronutrient supplementation [39], and of 60 g from balanced protein energy supplementation [40]. Our study will have power to detect a difference of 50 g, which is probably sufficient to be biologically relevant since mortality risk increases steeply with decreasing birth weight, especially below a 2500 g threshold [41, 42].

We aimed to obtain outcome data for at least 2220 infants per arm, and 8 880 infants in total assuming 20 clusters per arm (i.e. no loss of clusters), from women who could have >16 weeks exposure if they enrol early enough in pregnancy and whose birth weights are measured within 72 h.

We hypothesise that our interventions will improve infant nutrition, growth, stunting rates and the lifelong consequences of being born small for gestational age. For this reason the second primary outcome of infant weight-for-age z score (WAZ), and secondary outcomes of length-for-age and weight-for-length z scores, head circumference and maternal BMI and MUAC, are measured through endpoint nutrition clinics from June to October 2015. The age of trial infants at endpoint nutrition assessment will range from 1 to 22 months (most will be between 3 and 15 months).

We anticipate that, counting from the start of interventions on 13 Feb 2014, 190 permanently resident mothers per cluster will have participated by 31 Mar 2015 when birth weight measurement ceased and 210 by 20 Jun 2015 when nutrition endpoint follow-up began. We estimate that the second primary outcome will be obtained from at least 150 eligible infants per cluster born before clinics began on 20 June 2015 (70 % response rate). Assuming an ICC 0.01 then the design effect is 2.49 and effective sample size is 1205 per arm. This sample size provides 84 % power to detect as significant a difference between two arms of 0.12 in mean z score, if the z-scores have a standard deviation of one within each arm as expected. In Nepal we assume an average infant weight of around 6.5 kg at 6 months with standard deviation of 800 g for males, so for such infants a difference of 0.12 in Z-score represents about 96 g. Power to rank pairs of intervention arms of 80 % or more is expected should the difference in mean Z-score be 0.034 or greater.

#### Assignment of interventions and blinding

Selected clusters were allocated randomly to the four study arms. We stratified allocation on the basis of cluster size (population 4000–6399 versus 6400–9200) and accessibility. There were four strata: small and accessible, large and accessible, small and inaccessible and large and inaccessible. Random selection of clusters from the list of those eligible was undertaken via a transparent, public and participatory process. Key stakeholders such as District Public Health Office and District Development Committee officials, political leaders and NGO representatives were invited to a public meeting where the purpose of the trial was explained and stakeholder inputs gathered. Representatives were invited to draw the 80 study clusters and randomly allocate them using a ‘bingo’ or ‘tombola’ system. The process was video-recorded.

Although blinding of interventions for trial participants, care providers and field interviewers is not possible, outcome data analysts will be blinded to the identity of the study arms at both interim and final analyses. The data monitoring committee will also be blinded to the identity of the study arms during any interim analyses.

### Methods: data collection, management, and analysis

**Fig. 8:**
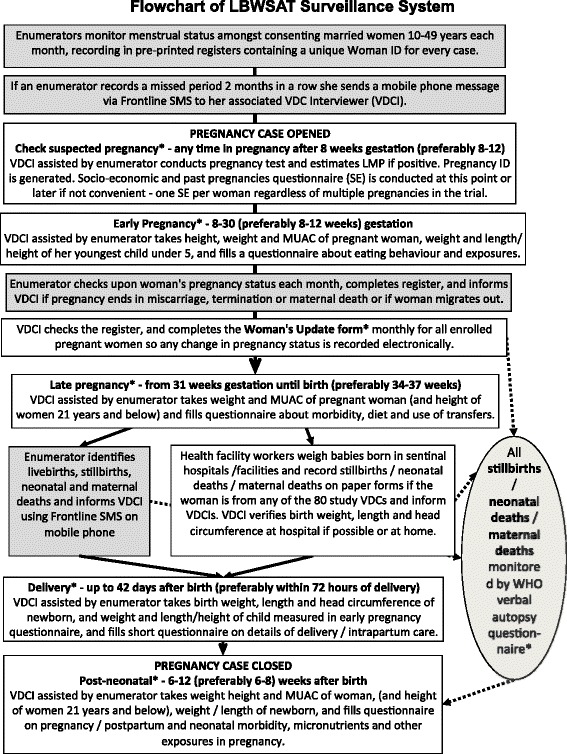
LBWSAT trial surveillance system for prospectively measured outcomes

**Fig. 9:**
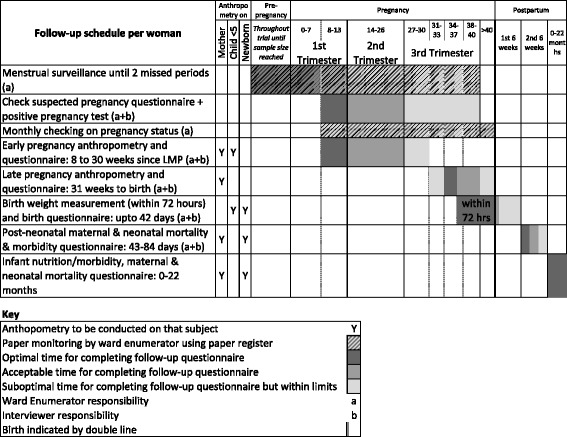
Follow-up schedule of pregnant women enrolled

A flowchart of the surveillance system is provided in Fig. 8 and a schedule showing how each woman enrolled in the surveillance system is followed up from pre-pregnancy to birth is provided in Fig. 9. Each enumerator records the names and other details of all married women aged 10–49 in her ward who consent to menstrual monitoring, in a pre-printed register. She checks the status of each woman monthly, and adds newly married women to her ledger. After identification of two missed menses in a row or a probable pregnancy, she alerts the VDC Interviewer to visit, validate the pregnancy and enrol the woman.

After validation of a pregnancy and completion of a ‘check suspected pregnancy questionnaire’ to ascertain probable LMP, each participant can be interviewed with the following questionnaires coded onto Android smartphones using the CommCare (www.commcarehq.org) data collection platform: socioeconomic information and past pregnancies (filled once per woman, regardless of multiple pregnancies in the trial); early pregnancy (8 to 30 weeks, usually before 26 weeks); late pregnancy (31 weeks to birth, usually after 34 weeks); birth (preferably within 72 h, but can be completed up to 42 days); post-neonatal (43–84 days after delivery, usually within 8 weeks).

Follow-up is challenged by absence of pregnant women when interviewers visit, in-migration of women to access transfers, enrolment in late pregnancy, overload and political disruption of interviewers work.

#### Collection of anthropometric data and questionnaires using smartphones

All surveillance questionnaires, sub-sample studies and quantitative intervention process monitoring are conducted with Android smartphones or push-button phone text-messaging. Three platforms are in place: FrontlineSMS (www.frontlinesms.com), Open Data Kit Collect (ODK – opendatakit.org), and CommCare. FrontlineSMS is a text messaging system used by 720 Ward Enumerators (WE) with low-cost push-button phones. Each WE is linked to an interviewer (VDCI) within the server and a SMS of just one character is automatically forwarded to the correct VDCI for them to take action. The VDCI contacts the WE by phone and arranges to interview the woman as soon as possible. ODK is an open access data collection platform that we are using with 539 nutrition mobilisers to record women’s group attendance, transfers provided and home visits on low-cost Android smartphones. It is also used for post-distribution monitoring by logistics team members and for endpoint nutrition follow-up clinic questionnaires. CommCare is the main platform for surveillance questionnaires and observation checklists, on Samsung Galaxy Y smartphones.

Questions and questionnaires use simple language, checked for cultural appropriateness and sensitivity and to ensure that they are unlikely to lead to biased responses. Because data collection is electronic, skips in questionnaires are handled automatically. Questionnaires were developed in English, translated into Nepali and Maithili, and back-translated into English for checking. Pre-tested electronic questionnaires are available in Nepali, Maithili and English.

#### Anthropometric equipment and training

We use Tanita BD590 scales for birth weight and children under five, Tanita solar weighing scales for mothers, and “Shorr boards” for height and length. We make the following anthropometric measurements:

- **Mothers:** height and weight in early pregnancy (8–13 weeks), weight in late pregnancy (34–37 weeks), at 6–8 weeks postpartum and during endpoint nutrition follow-up clinics. For mothers under 20 years of age, height is also taken at these times.
- **Infants:** weight, length and head circumference at birth (ideally within 72 h of delivery), weight and length within the first 42 days (if birth weight impossible) and/or at 6–8 weeks. Weight, length and head circumference at endpoint nutrition follow-up clinics when children are aged 0 to 22 months.

#### Supervision and duplicate measurements

Repeat readings are taken for all anthropometric measurements, with a third reading if the difference exceeds predefined ranges. Routine calibration of anthropometric equipment is carried out using standard procedures and tools. Equipment not meeting acceptable limits is replaced immediately.

#### Standardisation of anthropometric measurement across many observers

Monitoring Field Coordinators (MFCs) observe interviews conducted by VDCIs, and take duplicate readings to calculate intra- and inter-observer Technical Error of Measurement (TEM) on prospectively collected data. In addition, ten standardisation sessions were held for 82 field staff (66 VDCIs and 16 MFCs) in June-July 2014. Sessions required each data collector to take duplicate measurements (weight, height, MUAC for women; weight, length, head circumference for children) on 10 volunteers. Standardisation of 21 data collectors was conducted during training for endpoint nutrition follow-up clinics (for measures of weight, height and MUAC on 10 women). Intra- and Inter-observer Technical Error of Measurement (TEM) were calculated, and poor data collectors identified for re-training and additional field support.

#### Measurement of mortality and pregnancy outcome

Capture of pregnancy outcomes during main trial surveillance was undertaken by enumerators recording miscarriages, births and deaths in the paper registers used for menstrual monitoring. Changes in women’s status were then recorded on Android smartphones by VDCIs on a monthly basis. As political disturbance affected capture of these outcomes across some clusters, pregnancy outcomes, vital status of mother-child dyads at birth, 28 days and at the time of endpoint nutritional follow-up are recorded in the nutritional follow-up questionnaires.

### Statistical methods

Primary analysis of outcomes will be by intention-to-treat.

Birth weight (original primary outcome) can drop significantly, usually reaching its lowest within 48–72 h after birth, with a mean loss of around 5–7 % of initial birth weight [43–47], and we shall adjust for time after birth that measurements were taken and compare timing between arms. Analysis of the second primary outcome, weight-for-age z-score, will also be adjusted for age, despite the fact that age is accounted for within the calculations, in case of a different ‘age-trajectory’ of weight in the study area compared to the WHO standard population used to derive the scoring system. Primary analyses of the primary outcomes will each be based on available cases without imputation. Differences seen between arms in analysis of the second primary outcome (z-scores) will be expressed in terms of the corresponding difference in grams of birthweight.

Secondary analyses will also relate to the efficacy of the interventions. A per protocol analysis will compare outcomes for women who enrolled early in pregnancy and actually received the full intervention package of five or more transfers. A dose-response analysis will compare outcomes in women with different levels of exposure to the intervention. Analyses will be adjusted for measures of socioeconomic status, parity, timing of birth weight measurement and other variables identified at the time of finalising the analysis plan.

All analyses will be conducted using random effects models that account for clustering. In common with other cluster RCTs, there is a possibility of baseline imbalance of clusters in birth weight and socio-demographic predictors of birth-weight and possible imbalance between arms in predictive characteristics of the mothers and infants who provide outcome data. Adjustments will be made through regression analysis. Birth weights and infant z scores are expected to be normally distributed and will be analysed without prior transformation. The effect measure for the primary analyses will be a difference in birth weight or infant weight-for-age z score associated with intervention, with 95 % confidence interval, potentially adjusted for confounders. The primary comparisons will be with the control arm, but we shall attempt to rank interventions and calculate pairwise difference in effects. No formal adjustment for multiple comparisons will be made, but it will be acknowledged in the interpretation of the findings. We plan to undertake subgroup analyses on the basis of socioeconomic and food security categories to explore the equity impact of the interventions.

### Cost effectiveness economic evaluation

Assuming that one or more of the three interventions (PLA, PLA + food, PLA + cash) has an effect on the primary or secondary outcomes, the economic evaluation will, from the provider perspective, rank the interventions according to their cost effectiveness, and classify each intervention as highly cost effective, cost effective, or not cost effective using Nepali GDP per capita as the cost effectiveness threshold. Provider cost data will be collected from project financial accounts, staff time use interviews, vehicle logbooks and key informant interviews at health facilities. Primary outcomes will be birth weight and infant weight-for-age z score. Secondary outcomes include infant length-for-age z score, infant weight-for-length Z-score and prevalence of LBW.

The costs of the interventions will be estimated incrementally and prospectively using an ingredients approach, with current government provision as the comparator. In addition, the incremental cost and cost- effectiveness of adding food or cash transfers to women’s groups will be analysed from the provider perspective. Provider costs of caring for LBW infants and for the treatment of pregnancy complications will be proxied with data from household spending on care seeking in the private sector.

Start-up and programme costs will be identified separately from implementation costs, and monitoring and evaluation costs reported alongside intervention costs. Capital costs will be annualized and all costs will be adjusted for inflation, and reported in constant purchasing-power parity-adjusted international dollars. Incremental cost-effectiveness ratios (ICERs) will be calculated for the primary outcome measures, LBW, and LBW prevalence (cost per case of LBW averted). We will also utilise data from published sources, together with trial data to estimate the cost per year of life lost, death averted and DALY averted. This will allow winder comparison with other interventions but will be subject to great uncertainty, which will be transparently documented. Sensitivity analyses will assess the robustness of results to assumptions including joint cost allocation rules and discount rates. An analysis costing a 7 kg bag of super cereal as distributed in GoN programmes, as opposed to the 10 kg actually provided, may be undertaken to look at the potential cost of scale-up assuming a similar effect size can be achieved with a smaller ration. Data will be analysed using a Microsoft Excel costing tool specifically developed to enable comparative cost effectiveness analysis with other women’s group interventions [48, 49]. The results of the economic evaluation and costing of the interventions will be presented separately from the primary results of the trial to facilitate a full exposition of the methods and findings.

### Data monitoring

A data monitoring committee (DMC) has been formed in accordance with DAMOCLES guidelines. Since the magnitude of an interim effect that would be strong enough to consider stopping is large, we do not intend to ask the DMC to apply a stopping rule.

### Harms

We have no reason to believe that any of the interventions will cause harm. However, we shall track stillbirths, neonatal deaths, and maternal deaths, and record all miscarriages.

### Auditing

#### Bias in delivery or receipt of the interventions

Bias may arise if interventions systematically bypass the poorest, if there is imbalance between allocation arms, or through differential in-migration of poor women to access food and cash. We shall exclude temporary residents (in-migrators) of this kind from analyses.

#### Bias in ascertainment of outcomes

Capture of a higher percentage of home deliveries than hospital deliveries will skew the sample towards women who have home deliveries, who tend to be less educated, poorer and more likely to be multigravid (Sikorski et al.: Does Nepal’s Safe Delivery Incentive Programme redress inequalities in maternity care? Analysis of population surveillance data, under review).

#### Minimising bias

We shall examine response rates across socioeconomic quintiles and adjust for socioeconomic status or literacy and parity in analyses. We ensure that enumerators, FCHVs, NMs and group members are aware of the importance of including everyone within their wards, irrespective of caste, religion or disability status, and they actively encourage disadvantaged and marginalised women to attend. We used block randomisation stratified by cluster size and accessibility to minimise allocation arm imbalance after profiling clusters at the time of the population census.

## Discussion

Although other programmes have explored whether food or cash transfers generate dependency, this would be difficult to evaluate within the timescale of our trial and it seems an unlikely risk given that households only receive the transfer when women are pregnant over a seven-month period.

A concern is the effect of transfers upon migration of women between their marital and parental homes. Commonly, pregnant women, especially young primigravidae, relocate to their parental homes for late pregnancy and delivery. Provision of social transfers will undoubtedly increase in-migration of women whose parental homes lie in a food or cash transfer cluster. We permit any woman who claims to be resident to enrol for interventions, but will exclude her from the analysis when we identify her true status.

### Trial status

Data collection began in December 2013 and integrated interventions began on 13 Feb 2014. The birth weight capture rate was low from the start and political problems starting in September 2014 created further challenges to data collection from mothers with >16 weeks potential exposure to interventions. In light of the end of funding at November 2015, in January 2015 the trial management group decided to close birth weight capture on 31 March 2015 and to add infant weight for age Z-score as a primary outcome, collected at nutritional clinics in every cluster conducted between June and October 2015. The Nepal earthquakes of April and May 2015 also affected activities, but not intervention implementation to any significant level.

## Additional files

Additional file 1: Nutrient content of 150 g daily intake of WFP wheat-soya with sugar Super Cereal food supplement, in relation to the nutrients listed in the DFID TORs, FAO-WHO vitamin and mineral requirements [50]. Remarks contain notes about nutrients where appropriate. (PDF 324 kb)
Additional file 2: Information sheet showing different paragraphs inserted for different study arms. (DOCX 24 kb)
Additional file 3: Information sheet and consent form for menstrual monitoring or pregnancy follow-up. (DOCX 24 kb)

## Abbreviations

DFID: UK Government Department for International Development
DMC: Data Monitoring Committee
EDP: Extended Delivery Point
FCHV: Female Community Health Volunteer
FDP: Food Drop Point
FFC: Facilitation Field Coordinators
IFS: Institute of Fiscal Studies
IGH: Institute for Global Health
LBWSAT: Low birth weight South Asia Trial
MFC: Monitoring Field Coordinators
NM: Nutrition mobilisers
ODK: Open Data Kit
RCT: Randomised controlled trial
SARH: South Asian Research Hub
SCF: Save the Children
TSC: Trial Steering Committee
UCL: University College London
VDC: Village Development Committee
VDCI: Village Development Committee Interviewer
WE: Ward enumerator
WFP: World Food Programme
WG: Women’s group
