# Supplementary material for: Protocol of the Low Birth Weight South Asia Trial (LBWSAT), a cluster-randomised controlled trial testing impact on birth weight and infant nutrition of Participatory Learning and Action through women’s groups, with and without unconditional transfers of fortified food or cash during pregnancy in Nepal
Source: BMC Pregnancy Childbirth. 2016 Oct 21;16:320. doi: 10.1186/s12884-016-1102-x (PMC5073870; doi:10.1186/s12884-016-1102-x)
Supplement: Additional file 1: — Nutrient content of 150 g daily intake of WFP wheat-soya with sugar Super Cereal food supplement, in relation to the nutrients listed in the DFID TORs, FAO-WHO vitamin and mineral requirements [50]. Remarks contain notes about nutrients where appropriate. (PDF 324 kb) [file 12884_2016_1102_MOESM1_ESM.pdf]

Additional file 1. Nutrient content of 150g daily intake of WFP wheat-soya with sugar Super Cereal food supplement, in relation to the nutrients listed in the DFID TORs, FAO-WHO vitamin and mineral requirements[53]. Remarks contain notes about nutrients where appropriate.

**Nutrient**	**Per 100g dry weight of super cereal**	**per 150 g dry weight of super cereal**	**Unit**	**Notes on Super Cereal version 13**	**Nutrients as specified by DFID**	**DFID stipulation**	**% of DFID stipula-tion**	**UNIMAP multiple micro-nutrient supple-ment***	**% of UNIMAP formul-ation**
Energy	380	570	kcal	minimum	Kcal	350-500	114%		
Protein	17	17	%	17% (N x 6.25) minimum	Protein, % of energy	<25			
Fat	3.8	3.8	%	3.8% maximum	Fat				
Crude fibre	4.5	4.5	%	4.5% maximum	Crude fibre				
**Micronutrients from Vitamin/Mineral premix FBF-V-13**									
Vitamin A	3460	5190	IU	Dry Vitamin A Palmitate 250 Cold Water Dispersible Stabilized	Retinol, µg	800		800	NA
Vitamin D3	441.6	662.4	IU	Dry Vitamin D3 100 Water Dispersible	Vitamin D, IU	200	331%		
Vitamin E TE	8.3	12.45	mg	Stabilized Dry Vitamin E Acetate 50% Water Dispersible	Vitamin E, mg	10	125%	200	6%
Vitamin K1	30	45	µg	Dry Vitamin K1 5% Water Dispersible	Vitamin K, µg			70	64%
Vitamin B1	0.2	0.3	mg	Thiamine mononitrate	Thiamine, mg	1.4	21%		
Vitamin B2	1.4	2.1	mg	Vitamin B2 fine powder (Riboflavin)	Vitamin B2, mg	1.4	150%	18	12%
Vitamin B6	1	1.5	mg	Pyridoxine hydrochloride	Vitamin B6, mg	1.9	79%		
Vitamin C	90	135	mg	Ascorbic acid	Vitamin C, mg	70	193%	10	1350%
Pantothenic acid	1.6	2.4	mg	Calcium D Panthotenate	Vitamin B5, mg			1.4	171%
Folate, (DFE)	110	165	µg	Folic acid*	Folic acid, µg	400	41%	400	41%
Niacin (Vit B3)	8	12	mg	Niacinamide	Niacin, mg	18	67%	1.4	857%
Vitamin B12	2	3	µg	Vitamin B12 0.1% or 1% Spray Dried	Vitamin B12, µg	2.6	115%		
Biotin	8.2	12.3	µg	Biotin 1%	Vitamin B7, µg			1.9	647%
Iodine	40	60	µg	Potassium Iodide*	Iodine, µg	150	40%	65	92%
Iron (a)	4	6	mg	Ferrous fumarate fine powder	Iron, mg	30	33%	30	20%
Iron (b)	2.5	3.75	mg	Iron-sodium EDTA					
Zinc	5	7.5	mg	Zinc Sulphate Monohydrate	Zinc, mg	15	50%	2.6	288%
Carrier				Corn maltodextrin					
**Other minerals**									
Potassium	140	210	mg	Potassium Chloride with 0.5% silicon dioxide as anticaking agent, compliant with food chemical codex, min 90%<425 micron and min 60%<250 micron	Potassium, mg				
Calcium	362	543	mg	Dicalcium Phosphate Anhydrous, compliant with food chemical codex, min 95%<250 micron, total aerobic	Calcium, mg				
Phosphorous	280	420	mg		Phosphorous, mg				
Copper, mg					Copper, mg	2		15	
Selenium, µg					Selenium, µg	65		2	
Sodium, mg					Sodium, mg	minimum		150	
* Adequate dilution must be used in order to guarantee premix homogeneity						*as used in Osrin 2005			
