# Supplementary material for: Protocol of the Low Birth Weight South Asia Trial (LBWSAT), a cluster-randomised controlled trial testing impact on birth weight and infant nutrition of Participatory Learning and Action through women’s groups, with and without unconditional transfers of fortified food or cash during pregnancy in Nepal
Source: BMC Pregnancy Childbirth. 2016 Oct 21;16:320. doi: 10.1186/s12884-016-1102-x (PMC5073870; doi:10.1186/s12884-016-1102-x)
Supplement: Additional file 2: — Information sheet showing different paragraphs inserted for different study arms. (DOCX 24 kb) [file 12884_2016_1102_MOESM2_ESM.docx]

**Additional file 2. Information sheet showing different paragraphs inserted for different study arms**

**You will be given a copy of this information sheet.**

Title of Project: **The Low birth weight South Asia trial: a study into cost-effective interventions**

**Details of the study**

We are doing a research study in your district and we would like to invite you to participate. You do not have to take part if you don’t want to.

The research is being done by Mother and Infant Research Activities (MIRA) and University College London in the UK. MIRA has an office at Ramananda Chowk in Janakpur. The main office is in Kathmandu.

**What is the research?**

We are aiming to improve the weights of babies in Dhanusha and Mahottari districts. We are contacting 17,000 pregnant women to compare different ways to make their babies healthier, improve their growth and help them to do well in school. A good way of making babies healthier might be to make sure that their mothers have good diets during pregnancy. It isn’t clear what the best way to do this is: we want to find the best means of improving babies’ weight, so we are going to compare different ways of helping mothers to have better diets.

We are going to try out 4 ways of improving mothers’ diets in Dhanusha and Mahottari. Everyone will benefit from taking part.

1. In 20 VDCs we shall work with women’s groups organised by Female Community Health Volunteers and with other community members. Women from the community called nutrition mobilisers will also visit pregnant women at their homes. The women’s groups combined with home visits hope to improve women’s nutritional status during pregnancy so that they increase their intake of nutritious food, take more rest, practice better hygiene and hand washing and treat infections.
2. In 20 VDCs we shall work with women’s groups and home visits and give pregnant women a food supplement called Super Cereal.
3. In 20 VDCs we shall work with women’s groups and home visits and give pregnant women money to help them eat more nutritious food.
4. In 20 VDCs we shall support existing Government of Nepal programmes.

In all 80 VDCs, we shall provide training on mothers’ and babies’ nutrition, and on caring for small babies, for health workers in government facilities. Which group your VDC belongs to has been decided by a lottery system.

**Who are we inviting to participate in monitoring during pregnancy and after delivery?**

You can take part if you are a woman aged 10 to 49, live permanently in the study area and are pregnant.

**What will happen if you agree to take part?**

Now that we have confirmed that you are pregnant by taking a pregnancy test, the ward *ganak* will carry on visiting you, and we shall arrange 4 things:

1. First, we shall measure your height and weight and ask some questions about you and your household, previous pregnancies or children, what you have been eating, where you will be later in pregnancy, and where you think you will have the baby.
2. When you are in late pregnancy we shall weigh you again and talk to you about what you have been eating, what you have been doing during your pregnancy and where you plan to be for your delivery.
3. When your baby is born we shall either visit you at a health facility and/or at home. We shall weigh and measure your baby and ask you some questions about your delivery.
4. About six weeks after the birth of your baby we shall visit to ask you about your baby’s health, and weigh and measure you and your baby.
5. We may want to meet you at other times as well as these during your pregnancy to gather some extra, more detailed, information.

Sadly, and rarely, some babies do not survive. If this happens, we shall try to understand what happened by talking to you and your family. You don’t have to agree to this, but if you do the information we collect will help us to understand more about how these unfortunate things happen so as to try to develop ways of preventing them in the future.

**What will you get if you take part?**

As we said, there are 4 sorts of VDC.

*In cash plus women’s groups intervention clusters insert section A*

*In food plus women’s groups intervention clusters insert section B*

*In women’s groups intervention clusters insert section C*

*In control areas insert section D*

[SECTION A: Your VDC is one that gets **women’s groups, home visits and cash payments to pregnant women.**

**VDC with women’s groups and money during pregnancy**

Interviewers from MIRA will visit you during and after pregnancy.

Female Community Health Volunteers and Nutrition Mobilisers will invite you to take part in monthly meetings of the women’s groups. The meetings will be a chance to discuss issues that affect the health of you and your baby, to learn new things and to make friends with other women. You do not have to attend the meetings, but if you choose to during your pregnancy you may benefit by learning new things about nutrition and health and by being better prepared for the birth of your baby.

A nutrition mobiliser will come to visit you from time to time during pregnancy to talk to you about how to improve your diet and the health of yourself and your baby.

Up to 7 times during your pregnancy, we shall give you a monthly payment of NPR 750. In order to get the full amount you need to make sure that the *ganak* is aware of your pregnancy as early as possible.

**General conditions for distributing money in cash intervention VDCs:**

Only pregnant women who have had a positive pregnancy test or who are obviously visibly pregnant and who are resident in a cash intervention VDC will be eligible to receive transfers.

1. A pregnant woman will be considered resident in a cash cluster if they are planning to live there continuously for at least 3 months of their pregnancy and the VDC is the location of their sasural, their maiti or of rented accommodation in which they are living.
2. Women are eligible for a cash transfer once per month until their baby is born, up to a maximum of 7 transfers in total over 7 months amounting to a total of Rs 5,250 altogether.
3. A woman can enrol at any point in her pregnancy but if possible, to get the maximum number of possible transfers, it would be best if they could enrol within 8 weeks of their last menstrual period.
4. Only one transfer per month may be given to any enrolled pregnant women. If someone misses their transfer one month they cannot get more the next month.
5. A pregnant woman has to receive her transfer herself. Someone cannot receive it on her behalf.
6. A photo ID card will be required to receive transfers. Every enrolled pregnant woman should keep their ID card safely and show the ID card to receive cash.
7. The ID card will have a hole punched in it for every transfer received. The pregnant woman receiving the cash will also need to provide her photo ID card so that a nutrition mobilizer can scan it with a mobile phone. When a transfer is received a thumbprint or signature will also be required.
8. Transfers are given to the pregnant woman through women’s group meetings wherever possible. However if the enrolled pregnant woman does not attend the women’s group meeting, transfers may be given through home visits provided by the nutrition mobilizer.
9. If an enrolled pregnant woman permanently migrates to a similar cash intervention VDC, transfers will be continued. However transfer of cash will be stopped, if an enrolled woman migrates to VDC belonging to a different intervention arm.
10. The ward-level Nutrition Mobiliser is the key responsible person (contact person) for cash distribution activities in each ward.
11. If pregnant women are not found in their dwelling places/communities over the time that the NM is distributing cash in a month, those women will not be able to get cash that month. However, transfers may be resumed in the next month if the woman is still truly residing in the VDC.]

[**SECTION B:** Your VDC is one that gets **women’s groups, home visits and food supplement.**

**VDC with women’s groups with home visits and food supplement**

Interviewers from MIRA will visit you during and after pregnancy.

Female Community Health Volunteers and Nutrition Mobilisers will invite you to take part in monthly meetings of the women’s groups. The meetings will be a chance to discuss issues that affect the health of you and your baby, to learn new things and to make friends with other women. You do not have to attend the meetings, but if you choose to during your pregnancy you may benefit by learning new things about nutrition and health and by being better prepared for the birth of your baby.

A nutrition mobiliser will come to visit you from time to time during pregnancy to talk to you about how to improve your diet and the health of yourself and your baby.

Up to 7 times during your pregnancy, we shall give you a monthly 10kg ration of a specially-fortified flour called Super Cereal. In order to get the full amount you need to make sure that the *ganak* is aware of your pregnancy as early as possible.

**General conditions for distributing super cereal in food intervention VDCs:**

Only pregnant women who have had a positive pregnancy test or who are obviously visibly pregnant and who are resident in a food study VDC will be eligible to receive transfers.

1. A pregnant woman will be considered resident in a food cluster if they are planning to live there continuously for at least 3 months of their pregnancy and the VDC is the location of their sasural, their maiti or of rented accommodation in which they are living.
2. Women are eligible for a food transfer once per month until their baby is born, up to a maximum of 7 transfers in total over 7 months.
3. A woman can enrol at any point in her pregnancy but if possible, to get the maximum number of possible transfers, it would be best if they could enrol within 8 weeks of their last menstrual period.
4. Only one transfer per month may be given to any enrolled pregnant women. If someone misses their transfer one month they cannot get more the next month.
5. A pregnant woman has to receive her transfer herself. Someone cannot receive it on her behalf.
6. A photo ID card will be required to receive transfers. Every enrolled pregnant woman should keep their ID card safely and show the ID card to receive food.
7. The ID card will have a hole punched in it for every transfer received. The pregnant woman receiving the food will also need to provide her photo ID card so that a nutrition mobilizer can scan it with a mobile phone. When a transfer is received a thumbprint or signature will also be required.
8. Transfers are given to the pregnant woman through women’s group meetings wherever possible. However if the enrolled pregnant woman does not attend the women’s group meeting, transfers may be given through home visits provided by the nutrition mobilizer.
9. If an enrolled pregnant woman permanently migrates to a similar food intervention VDC, transfers will be continued. However transfer of food will be stopped, if an enrolled woman migrates to VDC belonging to a different intervention arm.
10. The ward-level Nutrition Mobiliser is the key responsible person (contact person) for food distribution activities in each ward.
11. If pregnant women are not found in their dwelling places/communities over the time that the NM is distributing food in a month, those women will not be able to get food that month. However, transfers may be resumed in the next month if the woman is still truly residing in the VDC.

**How much Super Cereal does a pregnant woman need to eat?**

When you receive each month’s ration you will be asked to eat 150 g of it every day. This is equivalent to 4 large thin rotis, 2 full bowls of halwa or porridge, or 15 laddus. If you can eat this much every day we believe it will be beneficial for your and your baby’s health. There will be enough food for you to share about half of it with your other family members, but you yourself need to eat *all* of the rest of the food in order to get the full benefit. If you share more than half of it and eat less than half of the ration yourself in one month, it may not be enough to improve your and your baby’s health.]

[SECTION C: Your VDC is one that gets  **women’s groups with home visits to pregnant women.**

**VDC with women’s groups and home visits to pregnant women**

Female Community Health Volunteers and Nutrition Mobilisers will invite you to take part in monthly meetings of the women’s groups. The meetings will be a chance to discuss issues that affect the health of you and your baby, to learn new things and to make friends with other women. You do not have to attend the meetings, but if you choose to during your pregnancy you may benefit by learning new things about nutrition and health and by being better prepared for the birth of your baby.

A nutrition mobiliser will come to visit you from time to time during pregnancy to talk to you about how to improve your diet and the health of yourself and your baby.

Interviewers from MIRA will visit you during and after pregnancy. If we can manage to keep in touch with you, you will be eligible to receive an ‘end of participation transfer’ of NPR1000 after your pregnancy has ended and all data has been collected.

This payment of NPR 1000 will only be available to pregnant women who have had a positive pregnancy test or who are obviously visibly pregnant and who are resident in a **government programmes or a women’s group only** study VDCs.

1. A pregnant woman will be considered resident in a study cluster if they are planning to live there *continuously* for at least 3 months of their pregnancy and the VDC is the location of their sasural, their maiti or of rented accommodation in which they are living.
2. A woman can enrol at any point in her pregnancy but if possible, it would be best if they could enrol within 8 weeks of their last menstrual period.
3. A woman who has participated in the study has to receive her transfer herself. Someone cannot receive it on her behalf.
4. A photo ID card will be required to receive the ‘end of participation transfer’. Every enrolled pregnant woman should keep their ID card safely and show the ID card to receive the payment.
5. The ID card will have a hole punched in it to show when the transfer has been received. The pregnant woman receiving the payment will also need to provide her photo ID card so that a nutrition mobilizer can scan it with a mobile phone. When the final NPR1000 transfer is received a thumbprint or signature will also be required.
6. Transfers will be given to the participating women by the interviewer on the last time they come to collect data, which will usually be around 6 weeks after the end of the pregnancy.
7. If an enrolled pregnant woman permanently migrates to a study VDC and participates in data collection there, the transfers can still be paid. However a transfer cannot be paid if an enrolled woman migrates to outside the study area.
8. If participating women are not found in their dwelling place around 6 weeks after pregnancy ends they may not be able to get the payment. ]

**[SECTION D:** Your VDC is one that gets **Government of Nepal Programmes.**

**VDC with Government of Nepal programmes**

MIRA Interviewers will visit you during and after pregnancy. If we can manage to keep in touch with you, you will be eligible to receive an ‘end of participation transfer’ of NPR1000 after your pregnancy has ended and all data has been collected.

This payment of NPR 1000 will only be available to pregnant women who have had a positive pregnancy test or who are obviously visibly pregnant and who are resident in a **government programmes or a women’s group only** study VDCs.

1. A pregnant woman will be considered resident in a study cluster if they are planning to live there *continuously* for at least 3 months of their pregnancy and the VDC is the location of their sasural, their maiti or of rented accommodation in which they are living.
2. A woman can enrol at any point in her pregnancy but if possible, it would be best if they could enrol within 8 weeks of their last menstrual period.
3. A woman who has participated in the study has to receive her transfer herself. Someone cannot receive it on her behalf.
4. A photo ID card will be required to receive the ‘end of participation transfer’. Every enrolled pregnant woman should keep their ID card safely and show the ID card to receive the payment.
5. The ID card will have a hole punched in it to show when the transfer has been received. The pregnant woman receiving the payment will also need to provide her photo ID card so that a nutrition mobilizer can scan it with a mobile phone. When the final NPR1000 transfer is received a thumbprint or signature will also be required.
6. Transfers will be given to the participating women by the interviewer on the last time they come to collect data, which will usually be around 6 weeks after the end of the pregnancy.
7. If an enrolled pregnant woman permanently migrates to a study VDC and participates in data collection there, the transfers can still be paid. However a transfer cannot be paid if an enrolled woman migrates to outside the study area.
8. If participating women are not found in their dwelling place around 6 weeks after pregnancy ends they may not be able to get the payment.]

***THE REST OF THE INFORMATION IS COMMON TO ALL ARMS***

**Are there any risks if you participate?**

We do not think that any harm will come to you from participating. However, we shall see you regularly to check that you are well, and if there is cause for worry we shall stop the study. It is possible that you might find sharing information about your periods or your pregnancy uncomfortable, or that you might find answering some of the questions about your pregnancy, delivery or antenatal care embarrassing or upsetting. You don’t have to answer any questions if you don’t feel like it. If you would like to talk to someone about the feelings generated by the questions, please contact any member of MIRA staff.

**Your information will be confidential**

All information you share with MIRA *ganaks* or interviewers will be kept confidential, which means that they are not allowed to tell anyone what you have told them. The information will be stored in registers and on computers, but they will be protected so that only authorised people will be allowed to look at them.

**Agreeing to take part**

Your participation is voluntary. Remember, it is up to you to decide whether to take part or not. Choosing not to take part will not disadvantage you in any way. If you do decide to take part you are still free to withdraw at any time and without giving a reason.

Antenatal, delivery or postnatal care at government health facilities will not be affected in any way, whether or not you decide to take part. If you decide to take part you will be given this information sheet to keep and be asked to sign or give a thumbprint on a form to say that you agree.

**More information**

If you have any questions, you can contact MIRA office. The addresses and phone numbers of the Janakpur and Kathmandu offices are provided on this information sheet.

**PLEASE DO NOT READ THE FOLLOWING SECTION TO THE PARTICIPANTS BUT EXPLAIN THAT FURTHER INFORMATION ABOUT THE TRIAL IS AVAILABLE ON THE INFORMATION SHEET, INCLUDING CONTACT DETAILS OF THOSE IN CHARGE**

**Contact information for the Low Birth Weight South Asia Trial:**

Mr. Bhim Prasad Shrestha, Trial Manager, Mother and Infant Research Activities (MIRA), Ramanand Chowk, Janakpur, Dhanusha. Tel 041-523371. Or,

Dr Naomi Saville, Senior Research Associate, University College London Institute for Global Health and Technical advisor to Mother and Infant Research Activities (MIRA), YB Bhawan, Thapathali (behind Imperial Finance building), GPO Box 921, Kathmandu Nepal. Tel: + 01-4101546.

**Data will be collected and stored in accordance with the UK Data Protection Act 1998.**

**Ethical approval**

The Nepal Health Research Council (NHRC) and University College London Research Ethics Committee have approved the study.
