# Supplementary material for: Protocol of the Low Birth Weight South Asia Trial (LBWSAT), a cluster-randomised controlled trial testing impact on birth weight and infant nutrition of Participatory Learning and Action through women’s groups, with and without unconditional transfers of fortified food or cash during pregnancy in Nepal
Source: BMC Pregnancy Childbirth. 2016 Oct 21;16:320. doi: 10.1186/s12884-016-1102-x (PMC5073870; doi:10.1186/s12884-016-1102-x)
Supplement: Additional file 3: — Information sheet and consent form for menstrual monitoring or pregnancy follow-up. (DOCX 24 kb) [file 12884_2016_1102_MOESM3_ESM.docx]

**Additional file 3. Information sheet and consent form for menstrual monitoring or pregnancy follow-up**

Information sheet for **women participating in menstrual monitoring** part of **“The Low birth weight South Asia trial”**  ***(translated into Nepali and Maithili)***

**You will be given a copy of this information sheet.**

Title of Project: **The Low birth weight South Asia trial: a study into cost-effective interventions**This study has been approved by the UCL Research Ethics Committee (Project ID Number: 4198/001) and by the Nepal Health Research Council (Project ID Number: 108/2012).

**Details of the study**

We are doing a research study in your district and we would like to invite you to participate. You do not have to take part if you don’t want to.

The research is being done by Mother and Infant Research Activities (MIRA), led by Prof DS Manandhar, and Prof Anthony Costello and Dr Naomi Saville from University College London in the UK. MIRA has an office at Ramanand Chowk in Janakpur. The main office is in Kathmandu.

**What is the research?**

We are contacting 17 000 pregnant women to compare different ways to make their babies healthier. We hope to improve the weights of babies in Dhanusha and Mahottari districts. We think that this might make them healthier, improve their growth and help them to do well in school. A good way of making babies healthier might be to make sure that their mothers have good diets during pregnancy. It isn’t clear what the best way to do this is: we want to find the best means of improving babies’ weight, so we are going to compare different ways of helping mothers to have better diets.

We are going to try out 4 ways of improving mothers’ diets in Dhanusha and Mahottari.

1. In 20 VDCs we shall work with women’s groups organised by Female Community Health Volunteers and with other community members. The groups hope to improve women’s eating during pregnancy so that they increase their intake of nutritious food. In these areas we shall give Rs1000 to women who take part in the trial after they deliver their baby.
2. In 20 VDCs we shall work with women’s groups and give pregnant women a food supplement.
3. In 20 VDCs we shall work with women’s groups and give pregnant women money to help them eat more nutritious food.
4. In 20 VDCs we shall support existing Government of Nepal programmes and give Rs1000 to women who take part in the trial after they deliver their baby.

In all 179 health facilities of Dhanusha and Mahottari, we shall provide training on mothers’ and babies’ nutrition, and on caring for small babies, for health workers in government facilities. Which group your VDC belongs to has been decided by a lottery system.

**Who are we inviting to participate?**

You can take part if you are a woman aged 13 to 49, live permanently in the study area, and could become pregnant.

**What will happen if you agree to take part in checking of whether you are pregnant or not?**

If you agree, we shall check whether you might be pregnant every month. We shall start to do this in Shrawan 2070 and will carry on until about Asad/ Shrawan 2072. Our ward *ganak* will record your name and address so we know who you are and where you live. Then she will visit you at home every month to check whether you might be pregnant by asking about your periods. She will keep what you say confidential and won’t tell your family members or neighbours anything you share with her.

If you think you might be pregnant, a MIRA team member will ask you to give a urine sample and will arrange a pregnancy test. If you turn out to be pregnant, we shall give you a photo ID card to show that you are involved in the study. Then we shall ask you questions and weigh and measure you and your baby several times. We shall tell you more about this in more detail if you become pregnant.

**What will you get if you take part in checking on your periods?**

As we said, there are 4 sorts of VDC. Your VDC is one that gets *[delete those that do not apply]*

**Government programmes only ☐**

**Women’s group only ☐**

**Women’s groups and food ☐**

**Women’s groups and cash ☐**

*If you do not become pregnant you will not get payments or food, but if women’s groups are working in your area you are welcome to attend. Women who become pregnant will all get some benefit, of either money or food, from participating.*

**Are there any risks if you participate?**

We do not think that any harm will come to you but it is possible that you might find sharing information about your periods or pregnancy uncomfortable or upsetting. You don’t have to continue to take part if you don’t feel like it. If you would like to talk to someone about the feelings generated by the questions, please contact a member of MIRA staff or Dr Saville.

**Your information will be confidential**

All information you share with MIRA *ganaks* or interviewers will be kept confidential, which means that they are not allowed to tell anyone what you have told them. The information will be recorded either on paper or entered onto mobile phones. The kind of information we shall keep about you will include your name, age or date of birth, address and the answers to questions that we ask you. The information will be stored in registers and on computers, but they will be protected so that only authorised people will be allowed to look at them.

**Ethical approval**

The Nepal Health Research Council (NHRC) and University College London Research Ethics Committee have approved the study.

**Agreeing to take part**

Your participation is voluntary. If you don’t want to take part, you can refuse without any problem. If you agree to participate and then change your mind at any time, please tell us and we shall stop visiting you. If you decide to take part you will be given this information sheet to keep.

**More information**

You should feel free to discuss the study with other people or ask us any questions.

If you have any more questions, you can contact Mr Bhim Prasad Shrestha, Trial Manager, Mother and Infant Research Activities (MIRA), Ramanand Chowk, Janakpur, Dhanusha. Tel 041-523371.

or

Dr Naomi Saville, Senior Research Associate, University College London Institute for Global Health and Technical advisor to Mother and Infant Research Activities (MIRA), YB Bhawan, Thapathali (behind Imperial Finance building), GPO Box 921, Kathmandu Nepal. Tel: + 01-4101546.

**Consent Form** for menstrual monitoring or for enrolment during pregnancy, follow-up between 8 weeks gestation and 84 days after birth and birth weight monitoring as part of the Low birth weight South Asia trial *(translated into Maithili and Nepali)*

Thank you for your interest in taking part in this research. Before you agree to take part, the person enrolling you in the research must explain the project to you. **Please complete this form after you have read and/or listened to the explanation about the research found in the Information Sheet.**

If you have any questions arising from the Information Sheet or explanation already given to you, please ask the researcher before you decide whether to join in. You will be given a copy of this Consent Form to keep and refer to at any time.

**Participant’s Statement**

I **___________________________________________________________________ (NAME)** (please print)

- have read / have had read out to me the notes written above and the Information Sheet, and understand what the study involves.
- agree that the research project named above has been explained to me to my satisfaction and I voluntarily agree to take part in this study.
- understand that I must not take part if I am not planning to stay at this address throughout my pregnancy and around the time of my delivery.
- consent to the processing of my personal information for the purposes of this research study.
- understand that such information will be treated as strictly confidential.
- agree that my non-personal research data may be used by others for future research. I am assured that the confidentiality of my personal data will be upheld through the removal of identifiers.
- understand that the information I have submitted will used in publications but also that confidentiality and anonymity will be maintained and it will not be possible to identify me from any publications.
- agree to be contacted in the future by researchers who would like to invite me to participate in follow-up studies.
- understand that if I decide at any time that I no longer wish to take part in this project, I can notify the researchers involved and withdraw immediately, and that neither I nor my antenatal or subsequent care will be affected negatively in any way if I do not want to participate.

**Participant’s signature or thumbprint:** __________________________ **Date:** ____________

**Researcher’s name** (please print): ________________________________ **Date:** ____________

**Researcher’s signature:** __________________________________________________________

This study has been approved by the UCL Research Ethics Committee (Project ID Number: 4198/001) and the Nepal Health Research Council (NHRC) (Project ID Number 108/2012).
